# Analysis of the structure and trend prediction of China’s total health expenditure

**DOI:** 10.3389/fpubh.2024.1425716

**Published:** 2024-09-24

**Authors:** Hong-yan Li, Rui-xue Zhang

**Affiliations:** School of Management, Shanghai University of Engineering Science, Shanghai, China

**Keywords:** total health expenditure, trend prediction, China, structural variation, gray prediction model, residents’ medical burdens

## Abstract

**Background:**

In the context of rapid economic and social development, there has been a continuous intensification of population aging, transformation of disease patterns, and wide application of new medical technologies. As a result, health expenditures in various countries have sharply soared. How to utilize limited medical resources to maximize the improvement of health levels has become a hot and challenging issue related to the well-being of all humanity. The relevant indicators of total health expenditure play a crucial role in monitoring and evaluating the fairness of health financing and health security in the region.

**Objective:**

This study explores the changes in the main expenses that constitute China’s total health expenditure and uses indicators related to health expenditure to observe the changes and future development trends of China’s health expenditure. Based on this, the utilization of China’s health expenditure is monitored to identify possible problems, and thereby targeted suggestions for promoting the development of China’s health and wellness cause are put forward.

**Methods:**

Based on the comparison of previous literature, this paper analyzes the changes and future development trends in China’s health expenditure by using the relevant indicators of China’s health expenditure through the structural variation analysis method and the gray prediction model.

**Results:**

The results show that the scale of government, social, and out-of-pocket health expenditures has continuously expanded, with social health expenditures becoming the main funding source for total health expenditures. The burden of medical expenditures on individuals has been further reduced. In the institutional method of total health expenditures, hospital expenditures account for about 60% of the total and are the main component. The expenditures of health administration and medical insurance management institutions are the main driving force behind the growth of total health expenditures. However, the proportion of health expenditures in China’s GDP is relatively low, so more investment is needed in the healthcare sector, and the burden of individual medical expenses also needs to be continuously reduced.

**Discussion:**

In the future, China should further increase its investment in the medical and health sector. Specifically, the government should persist in investing in fundamental medical and health services. Simultaneously, efforts should be made to establish a scientific cost control mechanism for pharmaceuticals and broaden financing channels for healthcare, such as accelerating the development of commercial health insurance.

## 1 Introduction

In China, the total health expenditure pertains to the value of economic resources expended by the entire society for the provision of medical and health services within a specific period (typically 1 year) in a country or region. It represents a significant indicator for gauging the financing level of health care and its utilization degree (1). It mirrors the degree of attention accorded by the government, society, and individual residents toward health under certain economic and social circumstances, the level of medical and healthcare costs borne, and the fairness and rationality of health financing. Therefore, this study aims to track the processes of fundraising, allocation, and usage of funds in the health system. It provides important data support for evaluating the sufficiency and sustainability of health fundraising, as well as the efficiency of fund usage. Additionally, it aims to measure the economic burden of medical treatment for the population. It is of great significance for optimizing the allocation of medical and health resources, ensuring the affordability of medical treatment for the masses, enhancing the fairness and accessibility of health services, and promoting the achievement of universal health. In recent years, China has initiated several relevant plans regarding health expenses. The “Healthy China 2030” Outline explicitly stipulates that by 2030, the proportion of out-of-pocket health expenditure in the total health expenditure will decline to approximately 25% (2). The “14th Five-Year Plan for National Medical Security” outlines the development goals of achieving a fairer and more inclusive basic medical security system, ensuring a more balanced burden-sharing among all parties, establishing guaranteed scope and standards that are better aligned with the level of economic and social development, and providing more accessible public services (3). The World Health Organization has even proposed that the proportion of total health expenditure in Gross Domestic Product (GDP) should be no less than 5%, and the proportion of out-of-pocket health expenditure in total health expenditure should range from 15 to 20% (4).

Due to the increasing demands for residents’ health, changes in population structure, and the rise in drug expenses, China’s total health expenditure has been continuously increasing. In 2022, China’s total health expenditure reached 8,532.749 billion yuan (equivalent to 1,268.603 billion US dollars at the current exchange rate), accounting for 7.05% of GDP. The *per capita* total health expenditure in China was 6,044.09 yuan (or 898.60 US dollars) (5). Among China’s total health expenditure, the proportion of individual health expenditure in the total health expenditure decreased to 26.89%, and the proportion of social health expenditure in the total health expenditure continued to ascend. Simultaneously, the government’s financing role for health has continuously strengthened. Despite the increase in total government health expenditures, the expenditure structure remains inadequately rational, and there has been no fundamental reversal of the situation where public medical and health resources are skewed toward treatment (6). In 2022, the proportion of health expenditure in the US GDP amounted to 16.63%. The proportion of health expenditure in the GDP of major OECD countries such as Japan, Germany, and the United Kingdom has exceeded 10% in the past few years (7). The medical and health expenditures in these countries are mainly derived from the government and society, accounting for approximately 90%, while out-of-pocket expenditures merely make up about 10%. In contrast to developed economies, the proportion of government health expenditures in China is excessively low and the proportion of out-of-pocket expenditures is relatively high, leaving the burden of residents’ medical expenses rather heavy.

Health expenditure has consistently been a topic of global preoccupation. In recent years, scholars across the world have predominantly centered their research on total health expenditure in terms of accounting outcomes, trend projections, and the analysis of influencing factors. In the majority of developed countries, health care expenditure has witnessed a sharp increase. Among them, the *per capita* expenditure on healthcare in the United States is twice that of any other developed country worldwide (8). Among the member states of the European Union, health expenditure is also one of the items with the fastest growth rate, and GDP and out-of-pocket health expenditure have been identified as the critical drivers of public health expenditure (9). However, as states in the United States are more homogeneous in terms of medical technology, consumer preferences, health policies, and the structure and general characteristics of the healthcare system, the convergence of healthcare costs among states in the United States might be quicker than among the European Union or OECD countries (8). Public health and health expenditures are significant for both developed and developing countries, but they are even more vital for the latter (10). For instance, Africa aspires to enhance health outcomes on the continent by increasing public health expenditure (11). Indian scholars contend that the ratio of public health expenditure to the country’s gross domestic product is a positively substantial predictor of healthcare infrastructure and human resources in rural areas of India (12). Health expenditures can result in the improved provision of healthcare opportunities, thereby reinforcing human capital, augmenting productivity, and boosting economic performance (10). In an economic environment featuring high levels of household consumption, employee wages, and physical capital investment, public health expenditure will considerably contribute to economic growth (13). Other scholars’ research has discovered that in the control of COVID-19, higher public health expenditure can shorten the time to reach the peak level of infection in the local area (14). Nevertheless, except the United States, all economies under examination have insufficient expenditures on healthcare. The insufficiency of expenditures is particularly acute in China, India, and the Russian Federation (15). So health expenditure is associated with the investment in medical care, the allocation of funds for health expenditure, and the equity of people’s health. There is a positive correlation between health expenditure and healthcare outcomes, but it is projected that in the upcoming years, health expenditure will further pose a challenge to financial sustainability (16).

**Figure 1:**
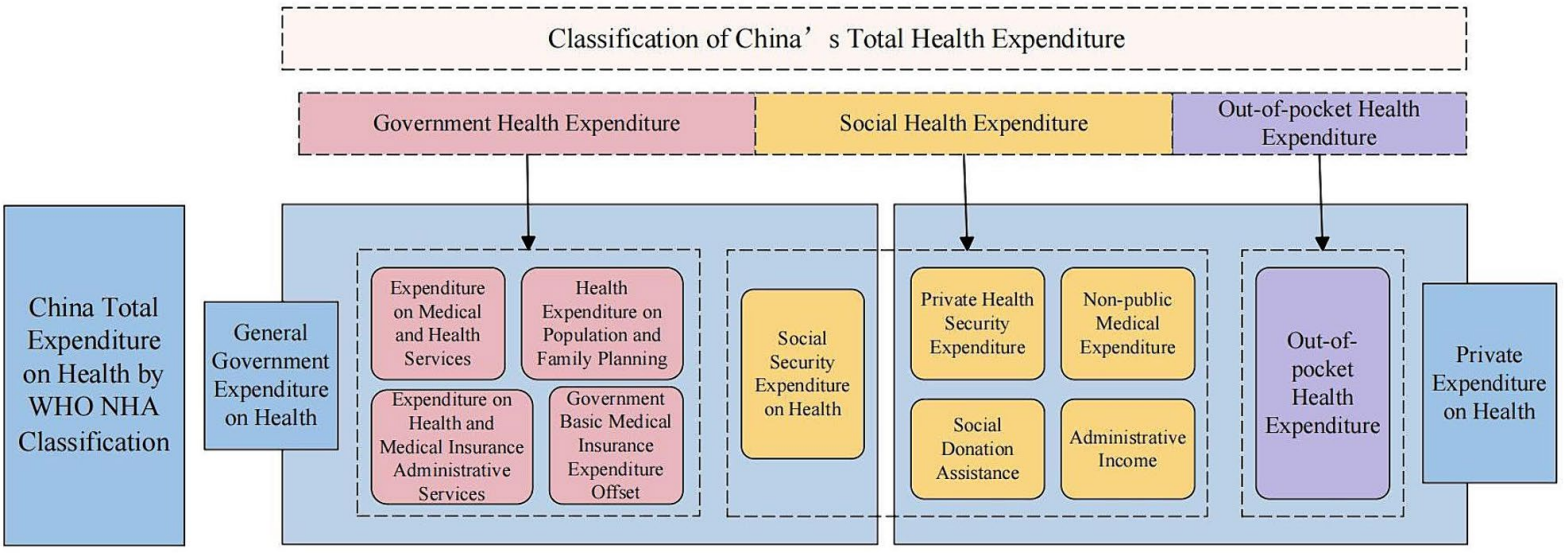
Classification of China’s total health expenditures.

Some studies show that the OECD’s System of Health Accounts (SHA) is commonly used as a basis for determining the measurement scope internationally (17). According to the International Classification for Health Accounts (ICHA) of the Statistical Abstract of the United States (SHA), total health expenditures can be divided into three categories: general government expenditure on health (GGHE), private expenditure on health (PHE), and the rest of the world, where the latter mainly refers to foreign aid expenditures, which usually come from international organizations, and the World Health Organization (WHO) generally includes it in the general government expenditure category (18). There are two ways to calculate the total health expenditures in China. One is the institutional approach, which defines total health expenditures as the sum of expenditures from public health institutions, health administration and pharmaceutical insurance management institutions, outpatient clinics, hospitals, pharmacies, and other sectors (19). The other is the source approach, which consists of government health expenditures, social health expenditures, and out-of-pocket health expenditures (20). According to the OECD classification of total health expenditures and the classification of total health expenditures in China, China’s total health expenditures can be classified as shown in Figure 1.

## 2 Materials and methods

### 2.1 Source of information

The data are sourced from *China Statistical Yearbook*, *Research Report on China’s Total Health Expenditure in 2020*, and *Statistical Bulletin on the Development of China’s Health and Wellness Undertakings in 2022*, and data such as China’s total health expenditure, government health expenditure, social health expenditure, out-of-pocket health expenditure, GDP, and health expenditure flowing to institutions like hospitals are extracted. Based on these data, structural variation analysis and trend prediction are carried out. Extract the specific composition data of diverse health expenses from the *Research Report on China’s Health Expenditure in 2020* as well as the personal health expenditure and drug expenses of certain developed countries, providing a reference basis for comparing with China’s health expenses and conducting in-depth analyses of the reasons for the structural variations of China’s health expenses.

The *China Statistical Yearbook* is an informative annual publication compiled and printed by the National Bureau of Statistics of China, comprehensively reflecting the economic and social development situation of China. It mainly includes a large amount of statistical data on the economy and society of the whole country, provinces, autonomous regions, and municipalities directly under the Central Government in the previous year collected in a certain year’s statistical yearbook, as well as the main statistical data of the country in important historical years and the past two decades. It is published by the National Bureau of Statistics every year and is China’s most comprehensive and authoritative comprehensive statistical yearbook. This article mainly selects the data on health expenditures and GDP in the *China Statistical Yearbook* from 2012 to 2022. *Research Report on China’s Total Health Expenditure in 2020* includes the main data of China’s total health expenditure from 1990 to 2019, the accounting results of health expenditure for each province, and briefly lists some historical materials since 1978 and foreign total health expenditure data. The *Statistical Bulletin on the Development of China’s Health and Wellness Undertakings in 2022* mainly describes the conditions of health resources and other aspects.

### 2.2 Methods

#### 2.2.1 Structural variation analysis method

As a dynamic data processing method, the structural change analysis method was mostly applied in resident consumption research in the early stage. It is also commonly used in the analysis of medical income and expense structure. It can comprehensively reflect the internal composition changes of medical expense structure and the overall characteristics of medical expense changes. This paper employs the method of structural variation analysis to study the measurement indicators of the structure of China’s total health expenditures, including the Value of Structure Variation (VSV), Degree of Structure Variation (DSV), Contribution rate of structural variation (CRSV), and driving force (21).

The Value of Structure Variation (VSV): $VSV=X_{i1}-X_{i0}$ . During a certain period, subtract the composition ratio at the beginning of each project from that at the end. If VSV > 0, the proportion of a certain project’s cost in the total cost increases, and this is a positive change; if VSV < 0, it is a negative change, and the situation is reversed.

Degree of Structure Variation (DSV): $DSV=\left|X_{i1}-X_{i0}\right|$ . This value always fluctuates between 0 and 1, reflecting the comprehensive change in the composition ratio of each project within a certain period. The size of the value reflects the degree of structural change; the larger the value, the greater the degree of change.

Contribution rate of structural variation (CRSV): $\mathrm{CRSV}=\left|X_{i1}-X_{i0}\right|/DSV\times 100\%$ . This value reflects the degree of influence of the changes in the proportion of each project in the overall on the overall cost structure; the larger the value, the greater the degree of influence.

Driving force = *CRSV* × *Project Growth Rate* × 100%. This value is to analyze the driving force of each item on the overall cost growth in combination with the direction of structural changes. The larger the value, the greater the driving force.

Among them, *i* represents the serial number of the expense item, 0 represents the beginning, 1 represents the end, $X_{i0}$ represents the composition ratio of a certain expense item to the total expense at the beginning, and $X_{i1}$ represents the composition ratio of a certain expense item to the total expense at the end.

#### 2.2.2 Gray prediction model

The gray prediction model is based on past and present known or uncertain information to construct a gray model (GM) and generate a hierarchical solution to obtain the generation function, thereby establishing a numerical sequence prediction for the target sequence (22). By forecasting the future trends of the system, it provides a basis for planning and decision-making. This forecasting method is designed for analyzing and modeling gray processes with limited information, sparse data, and concealed system laws. It has unique functions by generating and processing the original data sequence to weaken the randomness of the original data sequence and reveal the actual development laws of the system, thereby achieving the goal of forecasting.

This paper first analyzes the current situation and variations in China’s total health expenditures, and then, based on the initial time series of China’s total health expenditures from 2012 to 2022, it uses the gray system theory to establish a GM (1,1) model and conducts a test to predict and analyze the dynamic trend of China’s total health expenditures. The modeling process is as follows:

The original value sequence of total health expenditure for the constructed series is shown in Equation 1.

(1) $$X^{\left(0\right)}=\left\{X^{\left(0\right)}\left(1\right),X^{\left(0\right)}\left(2\right),\cdots \cdots ,X^{\left(0\right)}\left(N\right)\right\}$$

The cumulative value of total health expenditures is shown in Equations 2 and 3.

(2) $$X^{\left(1\right)}=\left\{X^{\left(1\right)}\left(1\right),X^{\left(1\right)}\left(2\right),\cdots \cdots ,X^{\left(1\right)}\left(N\right)\right\}$$

(3) $$X^{\left(1\right)}\left(N\right)=\left\{\begin{array}{l} X^{\left(0\right)}\left(1\right),N=1)\\ X^{\left(1\right)}(N-1+X^{\left(0\right)}\left(N\right),N=2,3,\cdots ,N \end{array}\right\}$$

Formulate the cumulative predictive equation for total health expenditures (Equation 4).

(4) $${\hat{X}}^{\left(1\right)}\left(t\right)=\left(X^{\left(0\right)}\left(1\right)-\frac{u}{a}\right)e^{-a\left(t-1\right)}+\frac{u}{a}$$

In Equation 4, *a* and *u* are the unknown parameters to be determined, which can be obtained from the gray parameter matrix:

(5) $$\hat{a}=\left[\begin{array}{l} a\\ u \end{array}\right]={\left(B^{T}B\right)}^{-1}B^{T}Y_{n}$$

In Equation 5, the sliding average matrix (*B*) and the data vector (*Yn*) are, respectively, defined in Equations 6 and 7. By substituting the obtained *a* and *u* into Equation 4, we get the equation expression of the cumulative predicted value of total health expenditure, and then by reducing it iteratively, we obtain the equation of the predicted value of total health expenditure for the “*t*” year, which is Equation 8.

(6) $$\left[\begin{array}{l} -\frac{1}{2}\left(X^{\left(1\right)}\left(2\right)+X^{\left(1\right)}\left(2\right)\right)\\ -\frac{1}{2}\left(X^{\left(1\right)}\left(2\right)+X^{\left(1\right)}\left(3\right)\right)\\ \vdots \\ -\frac{1}{2}\left(X^{\left(1\right)}\left(N-1\right)+X^{\left(1\right)}\left(N\right)\right) \end{array}\right]$$

(7) $$Y_{n}=\left[\begin{array}{l} X^{\left(0\right)}\left(2\right)\\ X^{\left(0\right)}\left(3\right)\\ \vdots \\ X^{\left(0\right)}\left(N\right) \end{array}\right]$$

(8) $${\hat{X}}^{\left(0\right)}\left(t\right)=\left\{\begin{array}{l} {\hat{X}}^{\left(0\right)}\left(t\right),t=1\\ {\hat{X}}^{\left(1\right)}\left(t\right)-{\hat{X}}^{\left(1\right)}\left(t-1\right),t\geq 2 \end{array}\right\}$$

Check the residuals and the relative error is given by Equation 9.

(9) $$\Delta \left(\varepsilon \right)=|\frac{X^{\left(0\right)}\left(t\right)-{\hat{X}}^{\left(0\right)}\left(t\right)}{X^{\left(0\right)}\left(t\right)}|$$

Average relative error $\bar{\Delta }\left(\varepsilon \right)=\frac{1}{n}\overset{n}{\underset{t=1}{\sum }}\Delta \left(\varepsilon \right)$ . When the average relative error is less than or equal to 0.2, the model passes the residual test. The model is diagnosed using the posterior difference test (23). Calculate the mean square errors of *X*^(0)^ and *ε*^(0)^(t) separately, denoted as *S*_1_ and *S*_2_, respectively. The posterior ratio *C* = *S*_1_*/S*_2_ is then calculated. The small error probability *p* < 0.6745*S*_1_ is also calculated.

This research examines China’s aggregate health expenditure structure, its trajectory, and forthcoming patterns through structural variation analysis and a gray prediction model. Currently, the application of structural variation analysis in China’s health expenditures primarily focuses on examining the composition of outpatient and inpatient expenses (24–26) and analyzing the distribution of health expenditures across different provinces and cities (27–29). Research on China’s health expenditures is limited to accounting-based analysis, without delving into a deeper understanding through structural variation analysis. The gray prediction model has found widespread utility for projecting health expenditure and anticipating future trends related to medical insurance funds within China.

## 3 Results

### 3.1 The composition of total health expenditure

#### 3.1.1 China total expenditure on health by source

The total health expenditure in China has generally demonstrated an upward tendency. Among the total health expenditure, the proportion of social health expenditure is the largest, while that of individual health expenditure has further decreased (Table 1). The total health expenditure has increased by 203% from 2012 to reach 85327.49 billion yuan, while the government health expenditure has increased by 15608.91 billion yuan, an increase of 185%. The social health expenditure has increased the most, rising to 38345.67 billion yuan. Out-of-pocket health expenditure has also increased, but the increase is the smallest, at 138%. The total health expenditure in China has shown an upward trend from 2012 to 2022, with the proportion of government health expenditure in total health expenditures declining from 29.99 to 28.17%. Although government health expenditure has shown a continuous growth trend, its proportion in total health expenditures has fluctuated within a small range. The proportion of social health expenditure in total health expenditures has shown an increasing trend, while the proportion of out-of-pocket health expenditure in total health expenditures has declined from 31.34 to 26.89%, reaching and falling below the 27% requirement mentioned in the “14th Five-Year Plan for National pharmaceutical Insurance.”

**Table 1:** Composition of total health expenditures in China from 2012 to 2022.

Year	Total health expenditure (billion yuan)	Government health expenditure		Social health expenditure		Out-of-pocket expenditure		Total health expenditure as share of GDP (%)
		Expenditure (billion yuan)	Share (%)	Expenditure (billion yuan)	Share (%)	Expenditure (billion yuan)	Share (%)	
2012	28119.00	8431.98	29.99	10030.70	35.67	9656.32	31.34	5.22
2013	31668.95	9545.81	30.14	11393.79	35.98	10729.34	33.88	5.34
2014	35312.40	10579.23	29.96	13437.75	38.05	11295.41	31.99	5.49
2015	40974.64	12475.28	30.45	16506.71	40.29	11992.65	29.27	5.95
2016	46344.88	13910.31	30.01	19096.68	41.21	13337.90	28.78	6.21
2017	52598.28	15205.87	28.91	22258.81	42.32	15133.60	28.77	6.32
2018	59121.91	16399.13	27.74	25810.78	43.66	16911.99	28.61	6.43
2019	65841.39	18016.95	27.36	29150.57	44.27	18673.87	28.36	6.67
2020	72175.00	21941.90	30.40	30273.67	41.94	19959.43	27.65	7.10
2021	76844.99	20676.06	26.91	34963.26	45.50	21205.67	27.60	6.72
2022	85327.49	24040.89	28.17	38345.67	44.94	22940.94	26.89	7.05

The data in the table is derived from the China Statistical Yearbook from 2012 to 2022 and the Research Report on China’s Total Health Expenditure in 2020.

Over the past decade, China’s total health expenditures have been fluctuating, with “increase and decrease” reflecting the warm process of meeting people’s needs and showcasing the solid steps toward building a healthy China. In the past decade, China’s total health expenditure as a percentage of GDP has increased from 5.22 to 7.05%, exceeding the WHO’s recommendation for medium-low income countries in 2010 (5–7%) and continuing to move toward a target of more than 7%.

#### 3.1.2 China total expenditure on health by provider

**Figure 2:**
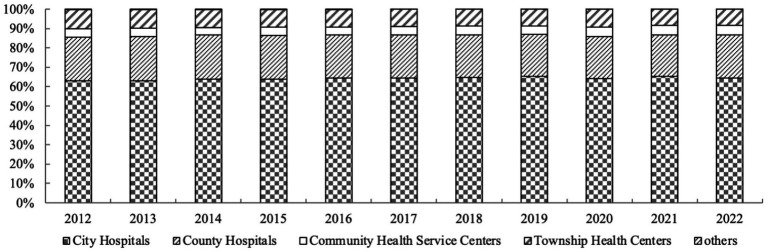
The share of expenditures for each institution in hospital organizations (2012–2022).

The total health expenditure in China is primarily allocated to hospitals, with a slight shortage of funding for primary care facilities (Table 2). In 2022, the total health expenditure (institutional method) in China directed 48,548.93 billion yuan toward hospital expenditures, accounting for 61.41% of the total expenditure. This figure was 0.74 percentage points lower than that in 2012, indicating overall fluctuations in funding allocation over the years. The expenditure directed toward public health institutions amounted to 502.568 billion yuan, accounting for 6.36% of the total expenditure. This percentage showed a consistent decrease from 2012 to 2019, followed by a slight increase to 6.56% in 2020 before declining once again. The proportion of expenditures in outpatient institutions is expected to remain stable within the range of 6–8%. The proportion of pharmacy retail expenditures has exhibited a slight downward trend, currently standing at 9.01%, while the proportion of expenditures in other institutions continues to rise within the total health expenditures. The proportion of expenditures flowing to hospitals has been relatively stable during the period from 2012 to 2022, with urban hospitals accounting for about 60% of the total expenditures and county hospitals about 20%. The expenditures of other primary health institutions account for a relatively small proportion (Figure 2).

**Table 2:** Institutional distribution of total health expenditures in China, 2012–2022 (%).

Year	Hospitals	Outpatient clinics	Pharmaceutical retail institutions	Public health facilities	Health administration and medical insurance management authorities	Others
2012	62.15	8.00	12.28	7.49	2.27	7.82
2013	62.33	7.43	12.45	7.38	2.29	8.12
2014	61.52	6.84	12.38	7.02	3.63	8.61
2015	61.73	6.74	12.47	6.56	3.34	9.15
2016	61.90	6.45	12.54	6.05	3.48	9.57
2017	62.59	6.64	11.73	5.85	3.20	9.98
2018	62.91	6.76	11.60	5.58	3.21	9.93
2019	63.55	6.91	11.67	5.47	3.34	9.05
2020	60.13	6.69	11.73	6.56	5.35	9.55
2021	63.67	6.92	8.86	6.40	4.49	9.65
2022	61.41	6.99	9.01	6.36	5.94	10.29

Source: The data in the table is derived from the Research Report on China’s Total Health Expenditure in 2020 and the Statistical Bulletin on the Development of China’s Health and Wellness Undertakings in 2022.

#### 3.1.3 China pharmaceuticals expenditure

Outpatient and retail pharmaceutical expenditures account for an increasing share of total health expenditures, while the share of hospital pharmaceutical expenditures has a downward trend. In 2022, the total expenditure on pharmaceuticals in China decreased by 13.46 percentage points to 21,275.81 billion yuan, accounting for 26.91% of the total health expenditure (institutional method). Of which, the retail pharmaceutical expenditure was 7,123.98 billion yuan, accounting for one-third of the total pharmaceutical expenditure. In 2020, retail pharmaceutical expenditures accounted for the largest share, but in 2021, they fell back to 30.91%, with the overall trend being an upward one for the proportion of retail pharmaceutical expenditures in total pharmaceutical expenditures. In 2022, the expenditures for outpatient pharmaceuticals and hospital pharmaceuticals were 8303.04 billion yuan and 5,848.79 billion yuan, respectively, accounting for 39.03 and 27.49% of the total pharmaceutical expenditures. Outpatient pharmaceutical expenditures have been increasing steadily, with the proportion of total pharmaceutical expenditures declining in 2013 and then rising again in subsequent years (Table 3). In contrast, inpatient pharmaceutical expenditures have fluctuated up and down, and the proportion of total pharmaceutical expenditures has been declining steadily, dropping by 7.68 percentage points compared to 2012.

**Table 3:** The composition of total pharmaceutical expenditure in China from 2012 to 2022.

Year	Pharmaceutical expenditure (billion yuan)	Outpatient pharmaceutical expenditure		Hospital pharmaceutical expenditure		Retail pharmaceutical expenditure		Pharmaceutical expenditure as share of THE (%)
		Expenditure (billion yuan)	Share (%)	Expenditure (billion yuan)	Share (%)	Expenditure (billion yuan)	Share (%)	
2012	11860.45	4082.74	34.42	4171.31	35.17	3606.40	30.41	40.37
2013	13307.70	4102.65	30.83	5043.48	37.90	4161.57	31.27	39.80
2014	13925.00	4203.43	30.19	5086.89	36.53	4634.67	33.28	37.20
2015	16166.34	5065.84	31.34	5674.11	35.10	5426.39	33.57	37.16
2016	17602.44	5471.30	31.08	6053.59	34.39	6077.55	34.53	36.32
2017	18203.00	5959.95	32.74	6037.84	33.17	6205.21	34.09	34.42
2018	19148.98	6286.22	32.83	6074.27	31.72	6788.50	35.45	32.37
2019	21116.82	7227.06	34.22	6490.30	30.74	7399.46	35.04	33.31
2020	20699.90	7093.89	34.27	5769.35	27.87	7836.66	37.86	30.98
2021	20395.63	7848.64	38.48	6242.58	30.61	6304.41	30.91	28.67
2022	21275.81	8303.04	39.03	5848.79	27.49	7123.98	33.48	26.91

Source: The data in the table is derived from the Research Report on China’s Total Health Expenditure in 2020 and the Statistical Bulletin on the Development of China’s Health and Wellness Undertakings in 2022.

### 3.2 Analysis of variation in the structure of China’s total health expenditure

#### 3.2.1 Analysis of structural variations of China’s total health expenditure by source

The total health expenditure in China has been increasing steadily, with individuals’ medical burdens further reduced. The variation in the structure of health expenditure sources in China (Table 4) indicates that, on the whole, government health expenditures and out-of-pocket expenditures have experienced negative variations, while social health expenditures have experienced positive variations. The overall structural variation in health expenditures is 15.54%. The structural variation in social health expenditures has the highest contribution rate, at 59.65%. On the other hand, out-of-pocket expenditures exhibit the smallest value of structural variation, while government health expenditures show the lowest contribution rate of structural variation. This indicates that out-of-pocket expenditures are decreasing, medical burdens are being alleviated to some extent, and the proportion of government health expenditures is showing a downward trend.

**Table 4:** Variations in the funding structure of total health expenditures in China (%).

Year	Government health expenditure		Social health expenditure		Out-of-pocket expenditure		DSV
	VSV	CRSV	VSV	CRSV	VSV	CRSV	
2012–2013	0.15	5.00	0.31	10.33	2.54	84.67	3.00
2013–2014	−0.18	4.35	2.07	50.00	−1.89	45.65	4.14
2014–2015	0.49	8.99	2.24	41.10	−2.72	49.91	5.45
2015–2016	−0.44	23.78	0.92	49.73	−0.49	26.49	1.85
2016–2017	−1.10	49.55	1.11	50.00	−0.01	0.45	2.22
2017–2018	−1.17	43.82	1.34	50.19	−0.16	5.99	2.67
2018–2019	−0.38	30.65	0.61	49.19	−0.25	20.16	1.24
2019–2020	3.04	50.00	−2.33	38.32	−0.71	11.68	6.08
2020–2021	−3.49	49.15	3.56	50.14	−0.05	0.70	7.10
2021–2022	1.26	49.80	−0.56	22.13	−0.71	28.06	2.53
2012–2022	−1.82	11.71	9.27	59.65	−4.45	28.64	15.54

From the value of structure variation, government health expenditures had positive variations in 2012–2013, 2014–2015, 2019–2020, and 2021–2022, while the overall structural variation value was negative. Social health expenditures had a negative structural variation value in 2019–2020 and 2021–2022, while they had a positive structural variation value in the other years. Out-of-pocket expenditures have shown a negative trend overall. From the perspective of the contribution rate of structural variations, it was observed that in 2012–2013, out-of-pocket expenditures made the largest contribution to structural variations, followed by social health expenditures. Government health expenditures were found to have the smallest contribution rate. The contribution rate of the structural change in social health expenditures has fluctuated, experiencing a decrease in 2014–2015. However, overall, there has been a gradual increasing trend compared to 10 years ago. This increase is on par with, or even slightly higher than, government health expenditures. In contrast, the contribution rate of out-of-pocket expenditures has steadily decreased over time.

#### 3.2.2 Analysis of structural variations in China’s total health expenditure by provider

The allocation of health expenditures is uneven, and there is still room for improvement in the expenditures of primary healthcare institutions. Overall, the structural variations in the allocation of healthcare expenditures in China indicate that the largest values of structural variation and contribution rates were observed in health administrative and insurance management institutions, at 3.67 and 29.86%, respectively, during the period from 2012 to 2022 (Table 5). The structural variation value of pharmaceutical retail institutions was the smallest, while the contribution rate of hospitals was the smallest. The degree structure variation of the allocation of healthcare expenditures in China was 12.29%, with the largest overall changes occurring in 2020–2021 and the smallest changes occurring in 2017–2018.

**Table 5:** Variations in the institutional flow structure of China’s total health expenditures.

Year	Hospitals		Outpatient clinics		Pharmaceutical retail institutions		Public health facilities		Health administration and medical insurance management authorities		Others		DSV
	VSV	CRSV	VSV	CRSV	VSV	CRSV	VSV	CRSV	VSV	CRSV	VSV	CRSV	
2012–2013	0.18	13.33	−0.57	42.22	0.17	12.59	−0.11	8.15	0.02	1.48	0.30	22.22	1.35
2013–2014	−0.81	22.13	−0.59	16.12	−0.07	1.91	−0.36	9.84	1.34	36.61	0.49	13.39	3.66
2014–2015	0.21	12.43	−0.10	5.92	0.09	5.33	−0.46	27.22	−0.29	17.16	0.54	31.95	1.69
2015–2016	0.17	10.63	−0.29	18.13	0.07	4.37	−0.51	31.88	0.14	8.75	0.42	26.25	1.6
2016–2017	0.69	26.74	0.19	7.36	−0.81	31.40	−0.20	7.75	−0.28	10.85	0.41	15.89	2.58
2017–2018	0.32	35.56	0.12	13.33	−0.13	14.44	−0.27	30.00	0.01	1.11	−0.05	5.56	0.9
2018–2019	0.64	32.32	0.15	7.58	0.07	3.54	−0.11	5.56	0.13	6.57	−0.88	44.44	1.98
2019–2020	−3.42	46.85	−0.22	3.01	0.06	0.82	1.09	14.93	2.01	27.53	0.50	6.85	7.3
2020–2021	−2.02	11.58	−1.31	7.51	−0.55	3.15	−0.93	5.33	3.08	17.66	−9.55	54.76	7.76
2021–2022	−2.26	49.02	0.07	1.52	0.15	3.25	−0.04	0.87	1.45	31.45	0.64	13.88	4.61
2012–2022	−0.74	6.02	−1.01	8.22	−3.27	26.61	−1.13	9.19	3.67	29.86	2.47	20.10	12.29

Regarding the value of structural variation, it was observed that public health institutions generally exhibited negative values, except a positive trend from 2019 to 2020. These institutions showed minimal fluctuations in expenditures. In contrast, health administration and medical insurance management institutions demonstrated predominantly positive structural variation values, accompanied by substantial expenditure variations. The proportion of expenditures allocated to hospitals showed a positive trend from 2012–2013 to 2014–2019, but exhibited a negative trend from 2019 to 2022, resulting in an overall small structural variation in the negative direction. On the other hand, the proportion of expenditures directed toward outpatient institutions displayed a negative structural variation value from 2012–2016 and 2019–2021, while showing positive trends in other years. However, the overall structural variation value remained negative. From the contribution rate of structural variations, the contribution rates of various expenditures to structural variations have fluctuated in 2012–2015, and in 2017–2022, the impact of hospitals and other institutions on the overall structural variations has been the largest. Additionally, by referring to Figure 2 and Table 3, we can see that the allocation of health funding in China is primarily concentrated in urban large hospitals, while there is a slight lack of resources allocated to grassroots healthcare institutions.

#### 3.2.3 Analysis of structural variations in China’s total health expenditure by provider

The fluctuation in hospital pharmaceutical expenditures has a significant impact on total health expenditures. On the whole, the structural variation values of pharmaceutical expenditures in China (Table 6) indicate that the structural variation value of outpatient pharmaceutical expenditures is the largest, at 4.61%, while the structural variation value of inpatient pharmaceutical expenditures is the smallest. However, the structural pharmaceutical contribution rate of inpatient pharmaceutical expenditures is the largest, while the structural variation contribution rate of retail pharmaceutical expenditures is the smallest. The structural variation degree of total pharmaceutical expenditures in China is 15.36%, and the overall variations in pharmaceutical expenditures for 2020–2021 are the largest, while the variations for 2015–2016 are the smallest.

**Table 6:** Variations in the structure of pharmaceutical expenditures in China (%).

Year	Outpatient pharmaceutical expenditure		Hospital pharmaceutical expenditure		Retail pharmaceutical expenditure		DSV
	VSV	CRSV	VSV	CRSV	VSV	CRSV	
2012–2013	−3.59	50.00	2.73	38.02	0.86	11.98	7.18
2013–2014	−0.64	15.92	−1.37	34.08	2.01	50.00	4.02
2014–2015	1.15	40.07	−1.43	49.83	0.29	10.10	2.87
2015–2016	−0.26	13.47	−0.71	36.79	0.96	49.74	1.93
2016–2017	1.66	50.00	−1.22	36.75	−0.44	13.25	3.32
2017–2018	0.09	3.10	−1.45	50.00	1.36	46.90	2.90
2018–2019	1.39	50.00	−0.98	35.25	−0.41	14.75	2.78
2019–2020	0.05	0.87	−2.87	50.00	2.82	49.13	5.74
2020–2021	4.21	30.29	2.74	19.71	−6.95	50.00	13.9
2021–2022	0.55	8.81	−3.12	50.00	2.57	41.19	6.24
2012–2022	4.61	30.01	−7.68	50.00	3.07	19.99	15.36

From the perspective of structural variation values, the structural variation values of outpatient pharmaceutical expenditures and retail pharmaceutical expenditures are mostly positive, and the structural variation values in pharmaceutical total expenditures are larger. From the perspective of the contribution rate of structural variation, it is evident that there have been significant changes in the contribution rates of each expenditure during this period. Of particular note is the substantial impact of hospital pharmaceutical expenditures on the overall structure, while outpatient pharmaceutical expenditures have had the smallest impact on the overall structure of total pharmaceutical expenditures.

### 3.3 The driving force for health expenditures from various expenditures

#### 3.3.1 The driving force of health expenditures by source

Social health expenditures have become the predominant component of total health expenditures, with government health expenditures demonstrating strong emergency response capabilities. Analysis of the driving forces behind health expenditure from various financing sources (Table 7) reveals that all factors contribute positively to the growth of total health expenditure. Among these factors, social health expenditure serves as the primary driving force, while government health expenditure plays a relatively smaller role. This indicates that social health expenditure has the greatest impact on the growth of total health expenditure, whereas government health expenditure makes the smallest contribution.

**Table 7:** The driving force for health expenditures from various expenditures from 2012 to 2022 (%).

Project	Growth rate	Contribution rate of structural variation	Driving force
Government health expenditure	11.05	11.73	129.57
Social health expenditure	14.35	59.83	858.60
Out-of-pocket expenditure	9.04	28.44	257.06
Hospitals	12.00	6.02	72.27
Outpatient clinics	7.78	8.22	63.91
Pharmaceutical retail institutions	12.36	26.61	328.88
Public health facilities	9.43	9.19	86.72
Health administration and medical insurance management authorities	17.04	29.86	508.76
Others	15.06	20.10	302.73
Outpatient pharmaceutical expenditure	7.36	30.01	220.79
Hospital pharmaceutical expenditure	3.44	50.00	171.89
Retail pharmaceutical expenditure	7.04	19.99	140.80

The contribution rate of government health expenditure structure variations has been growing steadily in recent years, with a particularly large increase after 2015, despite the continuous expansion of government health expenditures. The proportion of health expenditures has changed little and has shown a downward trend, mainly consisting of expenditures on healthcare services and health insurance subsidies, which together account for about 95%. The proportion of government expenditures on healthcare services has decreased from 52.01% in 2015 to 49.63% in 2019 and then increased to 58.22% in 2022. The proportion of government expenditures on health insurance subsidies has increased from 47.99% in 2015 to 50.37% in 2019 and then decreased to 41.78% in 2022. As a result, the government’s expenditure structure has changed (30). This shows that the government health expenditures can adjust its allocation proportion promptly to actively respond to major public health events.

**Figure 3:**
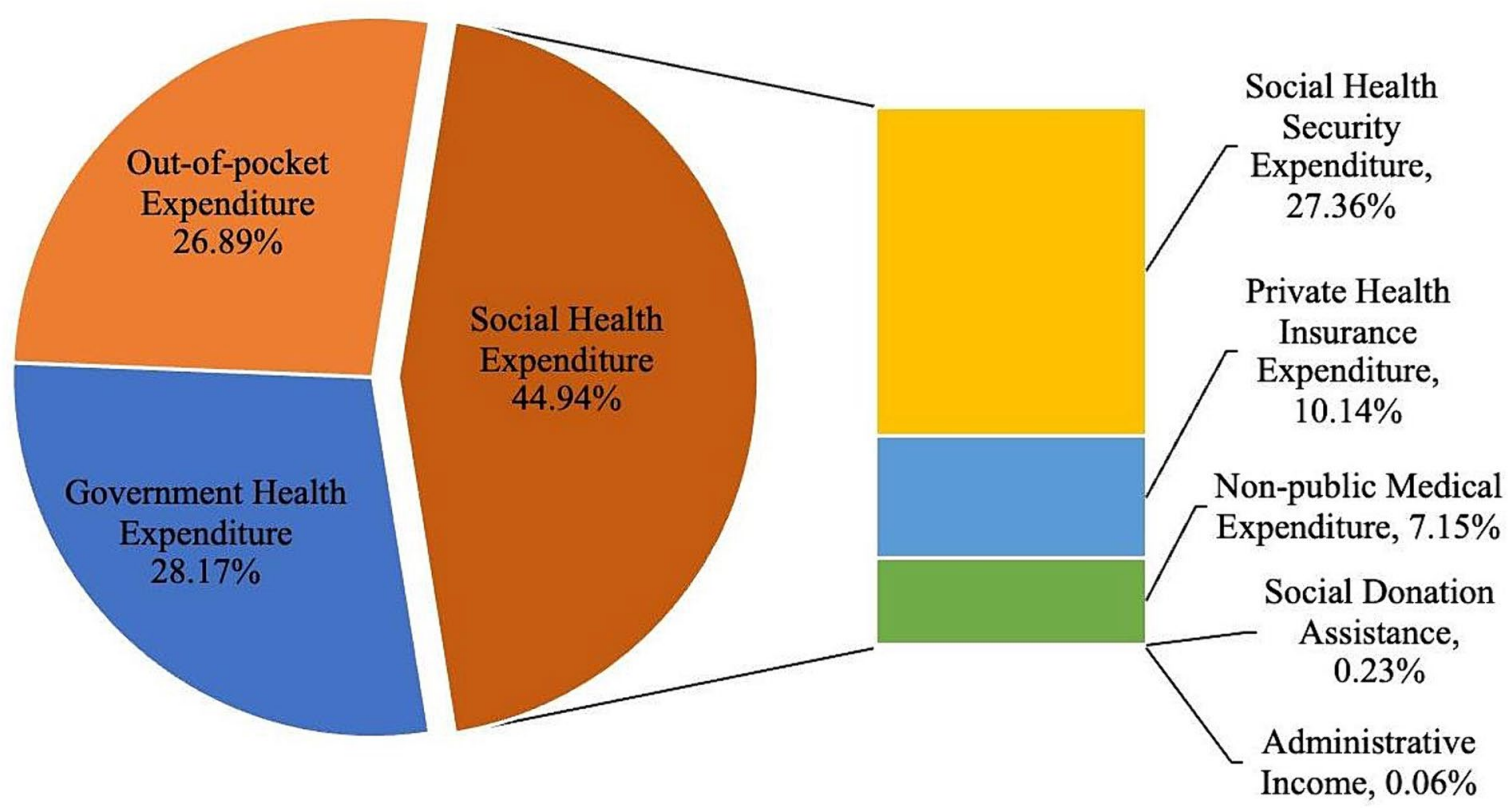
The composition of total health expenditures in China in 2020.

The contribution rate of social health expenditure to total health expenditure has been increasing steadily, and it has a powerful driving force for the growth of total health expenditure. Among social health expenditures, social security expenditures constitute half of the total health expenditures. Social security expenditures mainly denote the funds collected by various social medical insurance projects in the current year and do not incorporate government input (Figure 3). The funds collected are relatively large, so they also account for the largest share of social health expenditure. At the same time, due to the government’s robust support for commercial health insurance and the public’s growing awareness of healthcare, there has been a steady increase in premium income for commercial health insurance. This increase has contributed to social health expenditure and has enhanced the role of commercial health insurance in providing reimbursements, thereby increasing its share in total health expenditure. Meanwhile, social medical expenditure also plays a crucial role, making a significant contribution to the growth of total health expenditure. Direct investments from all sectors of society in various levels and types of healthcare institutions have driven the increase in social health expenditure. As a result, there has been a rising share of social health expenditure in total health expenditure.

#### 3.3.2 The driving force of health expenditures by provider

The growth of total health expenditure in institutional law is mainly driven by the expenditures of health administration and medical insurance management agencies. The various expenditures in the institutional flow have certain driving effects on the growth of total health expenditures (Table 7). The health administrative and medical insurance management institutions can raise more funds for medical insurance and other funds, and their expenditures have a greater driving force compared to other departments’ expenditures. At the same time, outpatient institutions primarily offer diagnosis and treatment services for outpatients and community family health care services. Due to their specific functions, the funds raised by these institutions are relatively less compared to other healthcare facilities. As a result, the expenditures of outpatient institutions have the smallest impact on driving force. Furthermore, the market scale of retail pharmaceutical institutions is continuously expanding and diversifying its development under the premise of standardized management, multi-channel circulation, and increasing chain rate year by year. As a result, their driving force for expenditures is substantial, enabling them to provide convenient pharmaceutical services for individuals or families in various regions. This can be explained by the fact that China’s medical insurance cause is experiencing rapid development, with a focus on strengthening the synergy between the medical, insurance, and pharmaceutical sectors. This development aims to promote the reform of the health and medical system in response to the needs of the people, ensuring that their demand for health services is consistently met.

#### 3.3.3 The driving force of pharmaceuticals expenditure

The outpatient department remains the mainstream for purchasing pharmaceuticals, and there is still considerable room for the development of retail pharmaceuticals. The pharmaceutical expenditures of various institutions have a strong driving effect on total health expenditures (Table 7). Among these factors, the driving effect of outpatient pharmaceutical expenditures is the most significant, while the impact of inpatient pharmaceutical expenditures on the structure of pharmaceutical expenditures is also substantial. On the other hand, retail pharmaceutical expenditures have the smallest driving effect. Although inpatient pharmaceutical expenditures have the largest impact on the structure of pharmaceutical expenditures, their driving effect is not as significant as that of outpatient pharmaceutical expenditures. This shows that outpatient pharmaceutical expenditures play an important role in the growth of total pharmaceutical expenditures, while the contribution of retail pharmaceutical expenditures to the growth of total pharmaceutical expenditures is small. Some scholars have found that the annual growth rate of pharmaceutical expenditures in China is 4.20%, far lower than the pharmaceutical expenditures growth rate during the “Thirteenth Five-Year Plan” period (31). The proportion of pharmaceutical expenditures in total health expenditures has also declined significantly, indicating that the era of “relying on pharmaceuticals to support medical services” is coming to an end. However, pharmaceutical income is still the largest item of outpatient income for public hospitals, and efforts need to be continued to control pharmaceutical expenditures. Controlling pharmaceutical expenditures should still be one of the important tasks for adjusting outpatient expenditure structure. In particular, within the outpatient population, patients with chronic diseases who require long-term medication often do not receive treatments and laboratory tests beyond regular checkups. Therefore, reducing pharmaceutical expenditures remains a crucial approach to controlling patients’ medical costs (32).

### 3.4 Projections for China’s total health expenditures

#### 3.4.1 Predict the overall expenditure on health in our nation

The total health expenditure and its share in GDP will continue to rise, with a gap remaining with developed countries. Using the four expenditures listed in Table 8 as predictor variables, after calculating the predicted values of the expenditures, this model passed the test, and the prediction results are scientific and reasonable. Finally, the ratios of government health expenditures, social health expenditures, and personal health expenditures to total health expenditures are calculated, and the ratio of total health expenditures to GDP is calculated through the model (Table 8). According to the results of the GM (1,1) model (Table 9), the government, social, and out-of-pocket health expenditures of all three sides have been continuously increasing, so the total health expenditures have been continuously increasing. It is expected that the total health expenditures in China will reach 205098.97 billion yuan by 2030. The proportion of out-of-pocket health expenditures to total health expenditures is expected to drop to 23.19%, achieving the goal set in the “Healthy China 2030” planning outline, which is 25%. Combining Table 5 shows that the proportion of government health expenditures and personal health expenditures is almost the same, and both are constantly decreasing, while the proportion of social health expenditures is gradually becoming the largest component of total health expenditures. In addition, it is expected that the proportion of total health expenditures to GDP will be 8.89% in 2030.

**Table 8:** Establishment and verification of GM (1,1) model.

Predictive indicators	GM (1,1) model	*C*	*P*	Result
Government health expenditure	$99982.37e^{0.0967\left(t-1\right)}-91550.39$	0.1580	1	Excellent
Social health expenditure	$101479.15e^{0.1221\left(t-1\right)}-91448.45$	0.1234	1	Excellent
Out-of-pocket expenditure	$113692.50e^{0.0886\left(t-1\right)}-104036.18$	0.0927	1	Excellent
GDP	$7233636.83e^{0.0794\left(t-1\right)}-6695057.83$	0.0824	1	Excellent

**Table 9:** Projections of health expenditures and financing structures.

Year	THE (billion yuan)	Government health expenditure		Social health expenditure		Out-of-pocket expenditure		GDP (billion yuan)	The Share of THE to GDP (%)
		Expenditure (billion yuan)	Share (%)	Expenditure (billion yuan)	Share (%)	Expenditure (billion yuan)	Share (%)		
2023	96939.02	26680.58	27.52	44689.89	46.10	25568.56	26.38	1323708.42	7.32
2024	107820.96	29388.53	27.26	50494.01	46.83	27938.43	25.91	1433157.49	7.52
2025	119951.20	32371.32	26.99	57051.93	47.56	30527.95	25.45	1551656.22	7.73
2026	133475.92	35656.86	26.71	64461.58	48.29	33357.48	24.99	1679952.85	7.95
2027	148558.69	39275.86	26.44	72833.55	49.03	36449.28	24.54	1818857.53	8.17
2028	165382.65	43262.17	26.16	82292.84	49.76	39827.65	24.08	1969247.35	8.40
2029	184152.87	47653.07	25.88	92980.65	50.49	43519.14	23.63	2132071.95	8.64
2030	205098.97	52489.63	25.59	105056.55	51.22	47552.79	23.19	2308359.49	8.89

**Figure 4:**
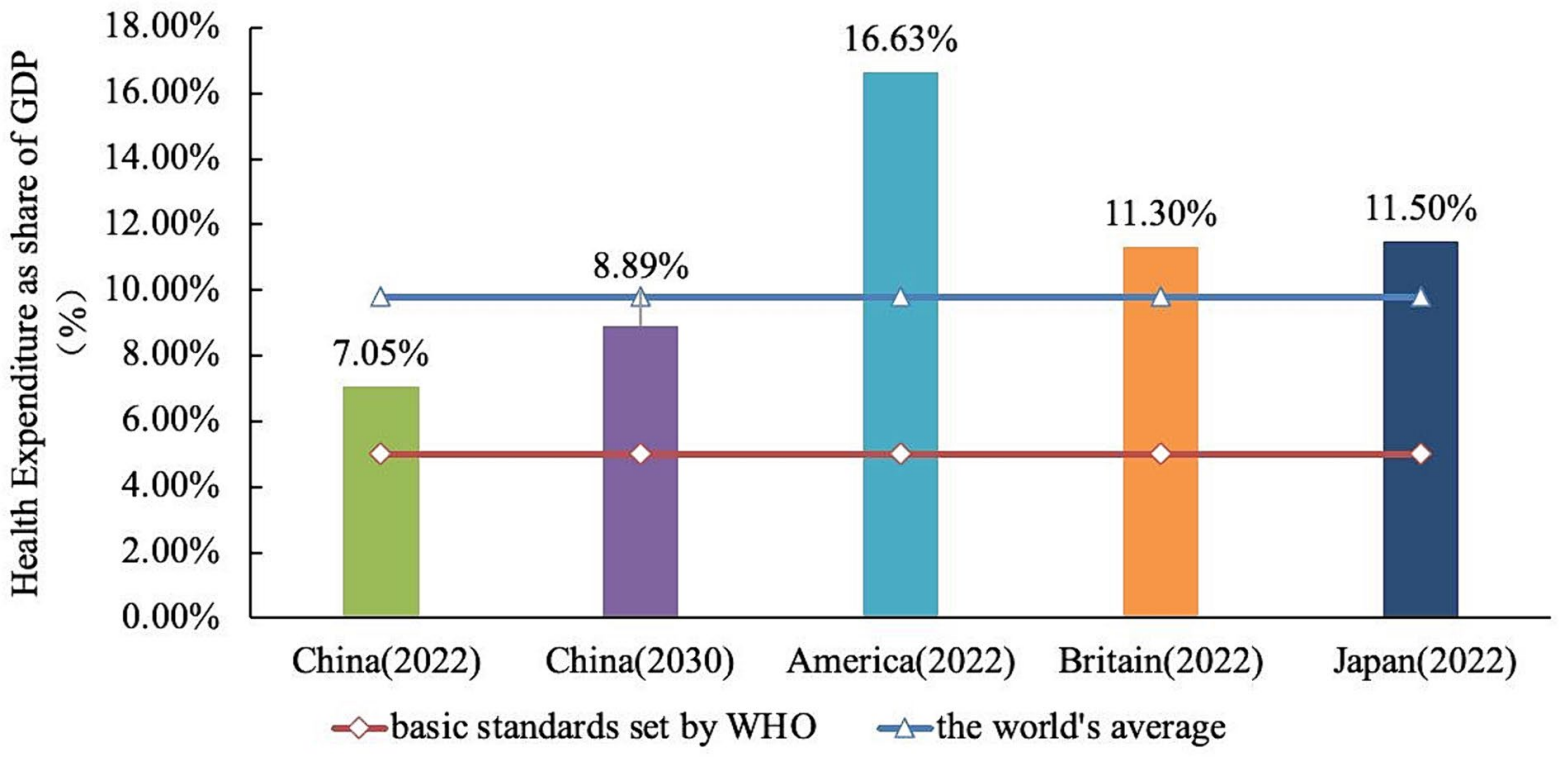
Comparison of healthcare expenditure as a percentage of GDP between some developed countries and China.

The proportion of out-of-pocket health expenditure in China has been steadily decreasing. However, there still exists a significant disparity when compared to the WHO’s initiative, which aims to reduce out-of-pocket health expenditure to 15% of the total health expenditure. This reduction is intended to minimize and eliminate catastrophic health expenditure and poverty resulting from illness. Meanwhile, in contrast to some developed countries of the OECD, the proportion of personal health expenditure in China relative to total health expenditure remains comparatively high. The proportion of out-of-pocket health expenditure in the majority of developed countries has been beneath 20%, and the proportion of total health expenditure to GDP in numerous developed countries has presently exceeded 10% (Figure 4). Hence, it becomes evident that the attention and investment in health expenditure still require further enhancement.

#### 3.4.2 Predict the pharmaceuticals expenditure on health in our nation

**Figure 5:**
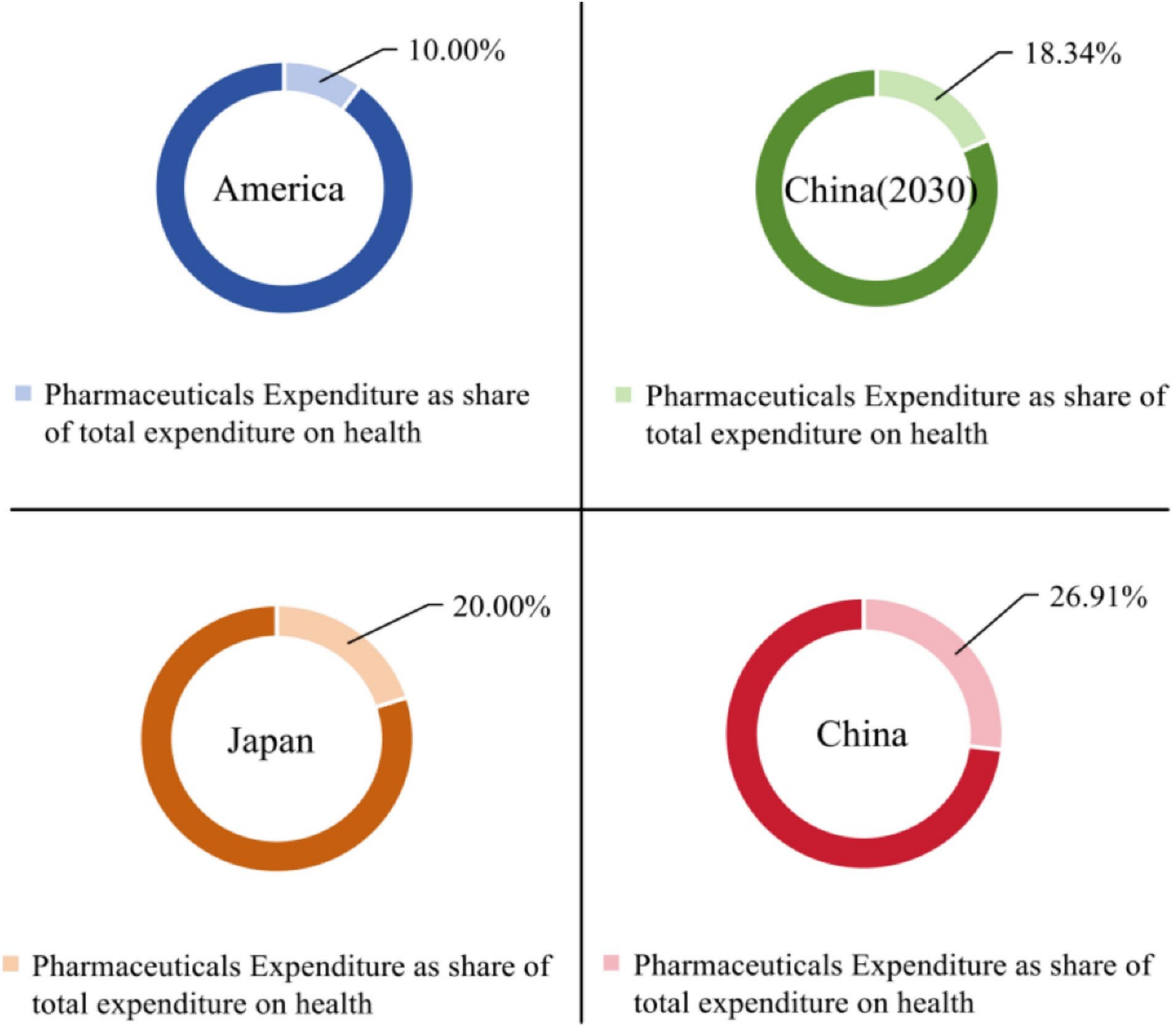
Comparison of the proportion of pharmaceutical expenditures in healthcare expenditures between China and selected developed countries.

The share of pharmaceutical expenditure in total health expenditure has decreased, but further efforts are needed to effectively control expenditures. The proportion of out-of-pocket health expenditures in China has been declining since 2015, mainly due to the deep reform of China’s medical and health system, which abolished pharmaceutical margins and adjusted medical service prices (33). The reform has achieved significant results. In 2017, China comprehensively launched the reform of public hospitals, and all public medical institutions abolished pharmaceutical margins (34), which has achieved significant breakthroughs in the pharmaceutical and healthcare system reform. Through forecasting, it can be seen that the proportion of pharmaceutical expenditures in total health expenditures will drop to 19.34% by 2030, and the effect of reducing residents’ medical expenditures through controlling pharmaceutical expenditures will be further manifested (Table 10). In addition, the “Guiding Opinion on Pilot Reform of Urban Public Hospitals” issued in 2015 proposed that the proportion of pharmaceuticals (excluding Chinese herbal pharmaceutical decoctions) in pilot city public hospitals should be reduced to around 30% in 2017 (35). However, the limited regulatory role of simply abolishing pharmaceutical margins in controlling medical expenditure, and the possibility of being offset by an increase in pharmaceutical, can only play a certain alleviating role. Controlling the pharmaceutical ratio at the same time will ensure sufficient price adjustment flexibility. In many developed countries, the proportion of healthcare costs spent on drugs has already fallen below 20% (Figure 5). This suggests that there is still significant potential for China to reduce its healthcare expenditures. Therefore, it is imperative to implement targeted measures aimed at cost control and alleviating patients’ financial burdens to achieve further reductions in healthcare costs.

**Table 10:** Predicted share of pharmaceutical expenditures in total health expenditures.

Year	Pharmaceutical expenditure (billion yuan)	Total health expenditures (billion yuan)	The share of pharmaceutical expenditures in total health expenditures
2023	23528.40	92069.52	25.56
2024	24696.45	101328.73	24.37
2025	25922.50	111519.12	23.24
2026	27209.40	122734.33	22.17
2027	28560.20	135077.43	21.14
2028	29978.06	148661.85	20.17
2029	31466.30	163612.41	19.23
2030	33028.43	180066.53	18.34

## 4 Conclusion

The continuous expansion of total health expenditure presents both a challenge and an opportunity for healthcare system reform in China. Firstly, as indicated by the above analysis, there is a rising trend in total health expenditure in China. Factors such as GDP and urbanization influence the growth of health expenditure in OECD countries, while improvements in health status, decline in mortality rate, and extended life expectancy have contributed to increased total health expenditure in Asian countries but reduced it in OECD countries (36). GDP, urbanization, and an aging population are also important factors contributing to the growth of health expenditures in China, a conclusion that has been confirmed by many scholars (37, 38). Furthermore, the out-of-pocket health expenditure among Chinese residents is on a decreasing trend. This can be attributed to the increasing contribution of social health expenditure to the total health expenditure. The shift toward social health expenditure as the primary contributor is a result of continuous improvements and advancements in China’s medical insurance system. These efforts are aimed at reducing the financial burden of medical expenses on residents. Despite increasing government health expenditure, greater fiscal input into the healthcare sector is still required in China, particularly for rural and grassroots healthcare.

Secondly, hospital expenses account for the majority of total health expenditures in China. Large urban hospitals have ample funds to equip themselves, possibly due to fiscal input, leading to the expansion of hospital scale. This results in increased medical expenses through higher numbers of hospitalizations and an increase in the average payment willingness of patients. And the subsidy from medical insurance will also raise medical expenses (39). Consequently, the funds allocated to the primary medical and health institutions are relatively less, and their expense growth rate is lower, and the proportion also shows a downward trend. If there are phenomena of excessive medical treatment in medical institutions, it will further lead to the unreasonable growth of medical expenses. Analysis of total health expenditure reveals that changes in expenses related to health administrative and medical insurance management institutions have the most significant impact on overall health expenditure. These changes are expected to drive the growth of health expenditure. The impact of hospital expenses and outpatient institution expenses on the overall changes in health expenditure is the smallest. Therefore, the function played by medical insurance is crucial, which can not only provide economic help to patients but also meet the adequacy of health expenditure financing in China.

Moreover, pharmaceutical expenses constitute a substantial portion of healthcare outlays for Chinese patients and significantly influence overall healthcare spending. They also contribute significantly to individual financial burdens associated with medical care. An examination reveals that pharmaceutical expenses in China have increased over time. However, their share relative to total healthcare expenditure has decreased due to regulatory measures introduced in 2016. These measures mandate pricing based on actual procurement cost plus a maximum markup limit set at 15% (35). These measures have curbed reliance on medications as a means to offset medical bills and facilitated rationalization of medical service charges through comprehensive healthcare reforms. Changes in hospital-based pharmaceutical expenses exert notable influence on overall variations in medication outlays while increases in outpatient fees drive aggregate pharmaceutical spending upwards. As a result, both outpatient and hospital-based medications play crucial roles. However, greater efforts are needed compared to developed nations to reduce reliance on medications for covering medical expenses. This leaves ample room for further development within the retail pharmacy sector.

Finally, the proportion of total health expenditures in GDP, which serves as the primary indicator for measuring the coordination between health expenditures and the national economy (40), reflects the level of social investment in healthcare and the degree of attention paid to residents’ healthcare. From the analysis of the results, it can be seen that the proportion of total health expenditures in GDP in China has been increasing steadily, and it has now entered the “7%” stage. It can also be predicted that the proportion of total health expenditures in GDP in China will continue to grow in the future. According to the data released by the World Health Organization (WHO), in 2021, the proportion of total health expenditures in GDP in China ranked 90th in descending order among WHO member countries (41), which still has development potential compared to the world average level.

## 5 Discussion

Firstly, it is necessary to clarify the policy areas covered by the health expenditure accounting results and determine the key tasks or priority problems to be addressed by policy analysis. Based on this, strike at the crux of the matter and set reasonable standards for the proportion of expenditure. The World Health Organization advocates that the broad government health expenditure ratio should be no less than 5% of GDP and the proportion of personal health cash expenditure in the total health expenditure of the country should be between 15 and 20% (42). If the proportion of personal health expenditure is lower than 15%, few families will suffer from catastrophic health expenditure (43). Therefore, it is recommended that the aforementioned international advisory indicators be established as the ultimate development goal. Suitable standards should be set for each stage, with a fluctuation range to address sudden public health emergencies. Efforts should also be made to ensure adequate investment in health and healthcare.

Secondly, the government should increase its financial support for preventive health services even further. It should also rapidly expand and balance medical resources, strengthen policies on talent training and salary incentives, and enhance the health management capacity of grassroots healthcare institutions. Additionally, efforts should be made to raise individual self-care levels in resisting diseases. The key to implementing the “Healthy China 2030” strategy is to shift the focus of investment from “disease treatment-centered” to “people-centered health,” and strengthen the service level and efficiency of grassroots healthcare institutions, to maximize the health benefits of government preventive spending. The reimbursement ratio for medical insurance can be moderately increased to reduce individual medical expenditures (44). However, it is important to consider the potential negative impact this may have on personal health investment, as well as the issues related to rising healthcare expenditure and fiscal reliance on medical insurance funds. Then, harmful health products such as alcohol, tobacco, and sugary drinks could be subjected to taxation or higher taxes. The revenue generated from these taxes can be allocated for health investment purposes, not only to address immediate health funding needs but also to mitigate long-term spending requirements by improving overall health conditions.

Moreover, China is a large country of generic pharmaceuticals, with about 95% of chemical pharmaceuticals being generics (45). However, due to the relatively backward development of China’s pharmaceutical industry, its generic pharmaceuticals compared with original research pharmaceuticals have a huge gap in quality and effect. Therefore, generic pharmaceuticals must pass the evaluation of consistency, be consistent with original research pharmaceuticals in terms of effective ingredients, dosage, safety, efficacy, and other aspects, and be allowed to be sold on the market. The level of consistency evaluation directly relates to the quality and therapeutic effect of the use of generic pharmaceuticals by patients, and stricter implementation of relevant policy opinions is needed to strictly control the entry threshold and ensure the effectiveness and safety of pharmaceuticals. Accelerating the pace of selection is necessary to improve the supply level of generic pharmaceuticals. There are still gaps in the targeted pharmaceuticals for certain diseases in China. It is essential to monitor the expiration dates of patented pharmaceuticals and expedite the process of updating the recommended list to encourage the production of generic pharmaceuticals, to meet the needs of patients. Promoting the assessment of consistency in generic pharmaceuticals aims to ensure that many generic drugs provide the same therapeutic effects and cost advantages as original research products. This will enable people to access high-quality and affordable medications, thereby reducing the economic burden of pharmaceutical use for residents.

Finally, the government should clarify its responsibilities, rationally control its financial input into the healthcare sector, and make adjustments based on real-time dynamics, ensuring the rationality of its input ratio. Encouraging active participation from all sectors of society in financing the healthcare sector and expanding social healthcare financing channels is crucial. Social healthcare financing plays a key role in ensuring the sustainable funding of China’s healthcare sector, and drawing on international experience can help promote diversified fundraising to meet the diverse healthcare service needs of different groups within the population. Accelerate the development of commercial health insurance and increase its proportion in total healthcare expenditures to increase the total social healthcare financing. Meanwhile, it is important to rationally allocate healthcare resources and stimulate the vitality of grassroots medical and health institutions. This can be achieved by promoting the principle of tiered medical treatment and building a pattern of “treating minor illnesses in communities, major illnesses in hospitals, and rehabilitation back in communities.” These efforts will lead to a continuous reduction in the proportion of out-of-pocket health expenditures in total healthcare expenditures, ultimately alleviating residents’ medical burdens.

## Data Availability

The original contributions presented in the study are included in the article/supplementary material, further inquiries can be directed to the corresponding author.
