# Corrigendum: Analysis of the structure and trend prediction of China's total health expenditure

**DOI:** 10.3389/fpubh.2024.1518663

**Published:** 2024-11-13

**Authors:** Hong-yan Li, Rui-xue Zhang

**Affiliations:** School of Management, Shanghai University of Engineering Science, Shanghai, China

**Keywords:** total health expenditure, trend prediction, China, structural variation, gray prediction model, residents' medical burdens

In the published article, there was an error in the Funding statement. The grant number was missing. The correct Funding statement appears below.

